# Empathy for others’ suffering and its mediators in mental health professionals

**DOI:** 10.1038/s41598-017-06775-y

**Published:** 2017-07-25

**Authors:** Hernando Santamaría-García, Sandra Baez, Adolfo M. García, Daniel Flichtentrei, María Prats, Ricardo Mastandueno, Mariano Sigman, Diana Matallana, Marcelo Cetkovich, Agustín Ibáñez

**Affiliations:** 1 Centro de Memoria y Cognición Intellectus, Hospital Universitario San Ignacio, Bogotá, Colombia; 2 Pontificia Universidad Javeriana, Psychiatry and Physiology Department, Bogotá, Colombia; 3 Grupo de Investigación Cerebro y Cognición Social, Bogotá, Colombia; 4 Laboratory of Experimental Psychology and Neuroscience (LPEN), Institute of Cognitive and Translational Neuroscience (INCyT), INECO Foundation, Favaloro University, Buenos Aires, Argentina; 5 National Scientific and Technical Research Council (CONICET), Buenos Aires, Argentina; 6 Departamento de Psicología, Universidad de los Andes, Bogotá, Colombia; 7 IntramedPortal www.intramed.net, Buenos Aires, CABA Argentina; 8 Faculty of Education, National University of Cuyo (UNCuyo), Mendoza, Argentina; 9 Universidad Torcuato di Tella, Laboratorio de Neurociencias, Buenos, Aires Argentina; 10 Pontificia Universidad Javeriana, Aging Institute Bogotá, Bogotá, Colombia; 11 Universidad Autónoma del Caribe, Barranquilla, Colombia; 12 Center for Social and Cognitive Neuroscience (CSCN), School of Psychology, Universidad Adolfo Ibáñez, Santiago de Chile, Chile; 13 Australian Research Council Centre of Excellence in Cognition and its Disorders, Sydney, Australia

## Abstract

Empathy is a complex cognitive and affective process that allows humans to experience concern for others, comprehend their emotions, and eventually help them. In addition to studies with healthy subjects and various neuropsychiatric populations, a few reports have examined this domain focusing on mental health workers, whose daily work requires the development of a saliently empathic character. Building on this research line, the present population-based study aimed to (a) assess different dimensions of empathy for pain in mental health workers relative to general-physicians and non-medical workers; and (b) evaluate their relationship with relevant factors, such as moral profile, age, gender, years of experience, and workplace type. Relative to both control groups, mental health workers exhibited higher empathic concern and discomfort for others’ suffering, and they favored harsher punishment to harmful actions. Furthermore, this was the only group in which empathy variability was explained by moral judgments, years of experience, and workplace type. Taken together, these results indicate that empathy is continuously at stake in mental health care scenarios, as it can be affected by contextual factors and social contingencies. More generally, they highlight the importance of studying this domain in populations characterized by extreme empathic demands.

## Introduction

Empathy is a complex construct, which entails feeling concern for others, sharing and comprehending their emotions, prompting motivation to help them^1, 2^. Rather than a unified domain, empathy represents a complex socio- cognitive competence encompassing various interacting components, such as affective sharing and perspective taking^3–5^. Moreover, empathy is a flexible capacity which becomes modulated by different cognitive, social, and contextual determinants^5, 6^. In addition to studies on healthy subjects^7–15^ and various neuropsychiatric populations^16–18^, a few reports have examined this domain focusing on professionals whose daily work particularly taxes empathic abilities, such as social workers, nurses, and physicians^19–21^. Building on this research line, the present population-based study assessed different dimensions of empathy in mental health workers (MHWs) and their relationship with socio-cognitive, demographic, and work-related factors.

Empathy skills in medical practice are continuously at stake. The work of physicians requires understanding the patients’ thoughts and emotional experiences, as well as effectively communicating their comprehension^19, 20, 22–24^. In this context, empathic skills emerges as a highly desirable trait^25^, since they foster trust^26, 27^, patient satisfaction^28^, diagnosis efficacy^21^, and, ultimately, treatment adherence and success^29–31^.

This proves particularly critical for mental health workers^32^. Psychologists and psychiatrists require highly empathic communication to understand and address their patients’ suffering. Karl Jaspers introduced empathy as a tool for psychopathological assessment more than a century ago^33^. Indeed, such a skill constitutes a cornerstone of all psychotherapeutic approaches, including psychodynamic^34, 35^, cognitive-behavioral^3^, and group psychotherapy^36^ approaches.

In medical contexts, empathy is modulated by several factors. For instance, this domain may be sensitive to the physicians’ moral profile, as low levels empathic concern predict utilitarian moral judgment in this population^37–39^. Furthermore, moral competence physicians and nurses seems to decrease as a function of age and years of experience^40, 41^. Thus, moral profile may also emerge as a key modulator of empathic dimensions in mental health workers.

Empathy also varies as a function of demographic factors. In physicians, these include gender^19^, expertise^13^, and workplace type^24, 42^. For instance, female medical workers showed higher scores than their male counterparts in self-reports of empathy. Concerning expertise, empathic sensitivity seems to decrease in the last stages of medical training^19, 43^, although experienced doctors can then recover their empathic behavior, arguably due to a reduction of personal distress^44, 45^. Arguably, medical workers with more than 10 years of experience, which is a criterion for being considered as experts or proficient practitioners, might present changes in clinical empathy^46, 47^. Finally, previous studies in general-physicians and nurses have explored empathy levels associated to workplace type^35, 36^. Those studies have shown that physicians working at inpatient environments report lower empathic reactivity than those in ambulatory environments. Arguably, medical professionals that work at ambulatory environments have more private and confident spaces, favoring the trust and welfare of patients^24, 42, 48^. In addition, medical professionals at inpatient contexts experience enhanced stress levels and have more risk of burnout syndromes, which could eventually affect empathy skills^24, 48–50^.

In sum, while clinical empathy seems sensitive to various factors, no study has explored this issue with a focus on mental health workers. Moreover, available results stem from relatively small samples completing self-report empathy questionnaires (which may be strongly biased by social expectations)^19, 51–53^. To address this issue, we conducted a population-based study aimed to (a) assess different dimensions of empathy for pain in mental health workers (MHWs) relative to a group of physicians (general-physicians) and non-medical workers; and (b) evaluate their relationship with relevant factors, such a moral profile, age, gender, years of experience, and workplace type. In particular, to circumvent the biases inherent to self-report measures or empathy scales, we used a validated empathy-for-pain task (EPT)^17, 18, 54–56^ tapping into cognitive, affective, and moral aspects of empathy. The EPT evaluates various dimensions of empathy in scenarios featuring intentional and accidental harm. The EPT employed here comprises 11 animated scenarios (4 intentional, 4 accidental, 3 neutral) involving two individuals. In this version, participants were asked to respond five questions for each scenario, i.e., (a) purpose comprehension (was the action done on purpose?), (b) empathic concern (how sad do you feel for the victim?), (c) degree of discomfort (how upset do you feel for what happened in the situation?), (d) intention to harm (how bad was the intention?), and (e) punishment (how much penalty does this action deserve?) (see Supplementary information for a further review of the EPT).

We hypothesized that MHWs, compared to both other groups, would exhibit higher empathy scores in all empathic domains. Furthermore, we predicted that the empathic profile of MHWs would be sensitive to factors such as morality, gender, years of experience. In addition, we predicted that workplace type (i.e., working at inpatient vs. ambulatory environments) could affect the empathic profile of MHWs. Considering that previous studies in general-physicians and nurses showed that workplace type modulates empathy, MHWs working at ambulatory environments should exhibit enhanced empathic skills relative to those working at inpatient environments. In short, we aimed to illuminate the interplay between empathy and various socio-professional factors in a population characterized by critical reliance on this domain.

## Materials and Methods

### Participants

The study comprised 1,109 individuals (567 women) with a mean age of 37.61 (*SD* = 12.5). All participants were professionals who accessed Intramed (www.intramed.net), an online portal designed for the healthcare community. The sample of MHWs (*n* = 377) was composed of 185 psychiatrists and 192 psychologists from 9 (nine) Latin-American countries (Argentina, Mexico, Colombia, Peru, Ecuador, Uruguay, Chile, Paraguay, Bolivia). The group of general-physicians included 402 individuals without medical residence, and the group of non-medical workers comprised 330 workers with no clinical experience who work in the fields of administration, economy, engineering, and social service. The participant’s age across groups ranged from 21 to 70 years. No significant differences were observed between groups in terms of gender (X^2^ (1) = 1.2, *p* > 0.1), age (*F* (1, 1035) = 0.22, *p* = 0.79), years of experience (*F* (1, 1035) = 0.41, *p* = 0.66), or workplace type (X^2^ (1) = 0.7, *p* = 0.39).

In a separate small-scale experiment, we have assessed another group of empathy domains with the interpersonal reactivity index (IRI), a widely used tool tapping different dimensions of affective and cognitive empathy^57^. In particular, we examined the extent to which empathic concern is related to personal distress and whether empathic concern and discomfort domains measured with the EPT coincide with the measures tracked by the IRI. To this end, we tested a group of 30 mental health workers, a group of 42 general-physicians, and a group of 28 non-medical workers.

All participants completed the survey and the experimental tasks in full and within a reasonable time (approximately 25 min). All subjects participated voluntarily by accepting an invitation posted on the main page of their Intramed profiles, and they gave informed consent in accordance with the Declaration of Helsinki by pressing an “I agree” button beneath an explanatory letter. Potential respondents were informed of the anonymity of their responses. All procedures performed in this study were approved by research committee and Ethics Committee of Javeriana University (Bogotá, Colombia) and Institute of Cognitive Neurology INECO (Buenos Aires Argentina).

All procedures in this study were conducted in accordance with the relevant guidelines and regulations of the Declaration of Helsinki.

### Instruments and procedure

On the first page of the online survey, participants reported their age, gender, occupation, workplace type (hospital or ambulatory service). Next, they completed a series of tasks, as described below.

### Empathy for pain task

We used a modified version of a previously reported EPT^16–18, 54–56^, which evaluates various dimensions of empathy in scenarios featuring intentional and accidental harm (for a further review of EPT procedure see Supplementary Information section 1.1).

### Moral judgment

Participants were also presented with two moral dilemmas^58, 59^, namely, an impersonal one (the standard trolley dilemma) and a personal one (the footbridge dilemma). We also included one non-moral dilemma for comparison purposes. In this dilemma participants were asked to choose whether to travel by bus or train given certain time constraints (see more details of moral judgment evaluation in Supplementary Information section 1.2).

### Interpersonal Reactivity Index

The IRI is a widely used tool for the multi-dimensional assessment of empathy^57^. This self-report instrument comprises 28 items answered on 5-point Likert scales ranging from “Does not describe me well” to “Describes me very well”. The tool is composed by four subscales, which explore different empathy domains, namely: (i) perspective taking (the tendency to spontaneously adopt the psychological point of view of others), (ii) fantasy (the tendency to imaginatively transpose oneself into the feelings and actions of fictitious characters in books, movies, and plays), (iii) empathic concern (“other-oriented” feelings of sympathy and concern for misfortunes befalling others), and (iv) personal distress (“self-oriented” feelings of personal anxiety and unease in tense interpersonal settings).

### Data analysis

Demographic data were compared among groups with ANOVA tests, except for categorical variables, which were analyzed through X^2^ tests. MANOVAs were run to explore group differences in empathic domains (each domain was considered as a dependent variable).

The ratings for each empathic measure were analyzed with factorial ANOVAS.

In addition, as in previous studies^16–18, 54, 55^, after MANOVA analyses, the ratings for each empathy measure (i.e., purpose comprehension, empathic concern, discomfort, intention to hurt and punishment) were independently analyzed through a factorial ANOVA. In each ANOVA we introduced condition (intentional harm, accidental harm and neutral situations) as a within-subject factor, and group (MHWs, general-physicians and non-medical workers) as a between-subject factor. In addition, each demographic factor (gender, age, years of experience, and workplace type) was introduced as a between-subject factor. We ran an independent analysis for each demographical factor (age, gender, workplace type, years of experience) to avoid mixed effects attributed to interactions between demographic factors, which correlate with each other. We reported only those contrasts between condition, group, and demographical factors that reached significance. When a significant interaction between group and condition was found, we examined between-group differences in ratings using Tukey’s HSD post-hoc test. Differences among conditions (intentional harm, accidental harm, and neutral situations) for each rating (purpose comprehension, empathic concern, discomfort, intention to hurt, punishment) were also examined with Tukey’s HSD post-hoc tests. Eta squared (n2) was used as a measure of effect size for significant effects. In the results section we reported Eta squared (n2) values for each significant p-value. Note that Eta squared is only available for ANOVAS main effects of interactions, but not for their post hoc comparisons (because the values are already considering the sum of squares obtained with the all ANOVAs combined effects).

Following a procedure reported in previous studies^18, 60^, we conducted multiple regression analyses to explore whether context factors (including gender, age, years of experience, workplace type) and responses to moral judgments partially explained performance on the EPT in each group. We considered as dependent variables all measures yielding group differences across conditions (intentional harm, accidental harm, neutral situations). Thus, we ran independent models for purpose comprehension, empathic concern, discomfort, intention to hurt, and punishment. Different demographical factors, including gender, age, workplace type, years of experience, and responses to personal and impersonal moral dilemmas, were introduced as predictors in all regression models.

For multiple regression analyses, multicollinearity of independent variables was assessed using variance inflation factors (VIFs) with a reference value of 3 before interpreting the final output^61^. Moral judgments, workplace type, and years of experience were introduced as dummy variables in the regression models. Thus, the moral judgment in the personal and impersonal dilemmas was coded with 0 when participants assigned utilitarian responses. Thus, positive values of beta were interpreted as a positive relationship between deontological judgments in moral dilemmas and the empathic measures. For workplace type, the variable was coded with 0 when participants had mainly worked in an inpatient environment and coded with 1 when participants had mainly worked at ambulatory environments. Negative values of beta were interpreted as greater empathic scores in ambulatory environments. Finally, participants were classified depending on whether they had more or less than 10 years of experience. This criterion was used considering evidence that medical workers change their empathic skills after 10 years of experience^46, 47^. Thus, for years of experience, the variable was coded with 0 when participants reported less than 10 years of experience. Negative values of beta were interpreted as greater empathic scores in subjects with more than 10 years of experience. Participants were classified depending on whether they had more or less than 10 years of experience. This criterion was used considering evidence that medical workers change their empathic skills after 10 years of experience^46, 47^. (for a further review of data analysis see Supplementary Information section 2).

## Results

### Group differences in empathy measures

A MANOVA analysis using the empathy domains as dependent variables (Purposely, EC, discomfort, intention to hurt and punishment) and group as between factor (MHW, general-physicians and non-medical professionals) revealed that the groups of subjects in each profession exhibited significant differences in empathy domains tracked with EPT Wilk’s (Wilk’s Lambda = 12.71) (*F* (36, 372) = 25.51, p < 0.0001, η2 = 0.21). This result allowed us to perform additional ANOVAS over each dependent variable of the EPT.

ANOVA analyses over purpose comprehension revealed no interactions between group and condition (*F* (4, 2216) = 1.12, *p* = 0.35) nor triple interactions between empathy condition, group, and demographic measures [gender (*F* (2, 2216) = 0.18, *p* = 0.94), age (*F* (16, 2216) = 0.63, *p* = 0.85), years of experience (*F* (2, 2216) = 1.02, *p* = 0.43), or workplace type (*F* (2, 2216) = 1.46, *p* = 0.13)].

**Figure 1:**
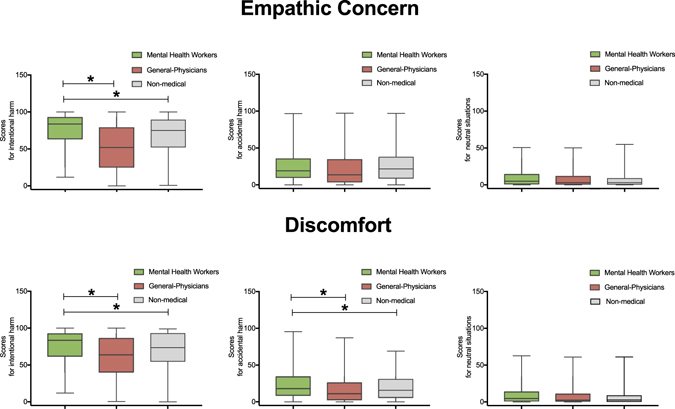
Ratings in empathic concern and discomfort domains in each group. This graph depicts ratings in two empathic measures (Empathic concern and Discomfort) by each condition (intentional and accidental harm and neutral situations) by each group. Stars indicate significant differences (*p* < 0.01).

Regarding empathic concern, results revealed a significant interaction between group and condition (*F* (4, 2216) = 16.10, *p* = 0.001, η2 = 0.1) for intentional harm. A post-hoc analysis (Tukey HSD, *MS* = 108.76, *df* = 2216) showed that MHW provided higher empathic concern ratings for intentional harm than general-physicians group (*p* < 0.01) and non-medical workers (*p* < 0.01). No other differences were observed in scores for accidental harm and neutral situations between groups (all *p*s > 0.4) (see Fig. 1).

Results for this empathy measure also revealed a triple interaction between empathy condition (intentional harm, accidental harm, neutral situations), group (MHWs, general-physicians, non-medical workers), and workplace type (*F* (2, 2216) = 2.46, *p* = 0.01, η2 = 0.06). A post-hoc analysis (Tukey HSD, *MS* = 108.76, *df* = 2216) showed that MHWs provided higher empathic concern ratings for intentional harm in environments of ambulatory practice than in hospitals (*p* < 0.01). No differences due to workplace type were observed in general-physicians group or non-medical workers. No interactions were found when experience factor was analyzed.

As regards discomfort ratings, a significant interaction was observed between group and condition (*F* (4, 2216) = 11.59, *p* < 0.01, η2 = 0.11). A post-hoc analysis (Tukey HSD, *MS* = 108.76, *df* = 2216) showed that MHWs provided higher ratings for intentional harm than general-physicians (*p* < 0.01) and non-medical workers (*p* < 0.01). Moreover, post-hoc analysis revealed that MHWs provided higher scores for accidental harm than general-physicians (*p* < 0.05) and non-medical workers (*p* < 0.05). No differences were observed between the latter two groups (*p* = 0.23 for intentional harm, and *p* = 0.33 for accidental harm) (see Fig. 1). No between-group differences were observed in scores for neutral situations (all *p*s > 0.3).

For the discomfort measure, an interaction emerged between condition (intentional harm, accidental harm and neutral situations), group (MHWs, general-physicians, non-medical workers), and years of experience (*F* (2, 2216) = 5.46, *p* = 0.01, η2 = 0.07). A post-hoc analysis (Tukey HSD, *MS* = 108.76, *df* = 2216) showed that in the MHWs, those professionals with more than 10 years of experience provided lower discomfort ratings than workers with less than 10 years of experience (*p* < 0.01). Post-hoc analyses did not reveal other significant effects (no differences were observed for general-physicians or non-medical group (all *p*s > 0.3). In addition, a triple interaction was observed between condition, group, and workplace for this measure (*F* (2, 2216) = 4.16, *p* = 0.01, η2 = 0.08). A post-hoc analysis (Tukey HSD, *MS* = 108.76, *df* = 2216) showed that workers in MHWs provided higher discomfort ratings for intentional and accidental harm in ambulatory environments than in hospitals (all *p*s < 0.01). Post hoc analyses did not reveal other significant effects (no differences were observed for general-physicians or non-medical workers (all *p*s > 0.5).

**Figure 2:**
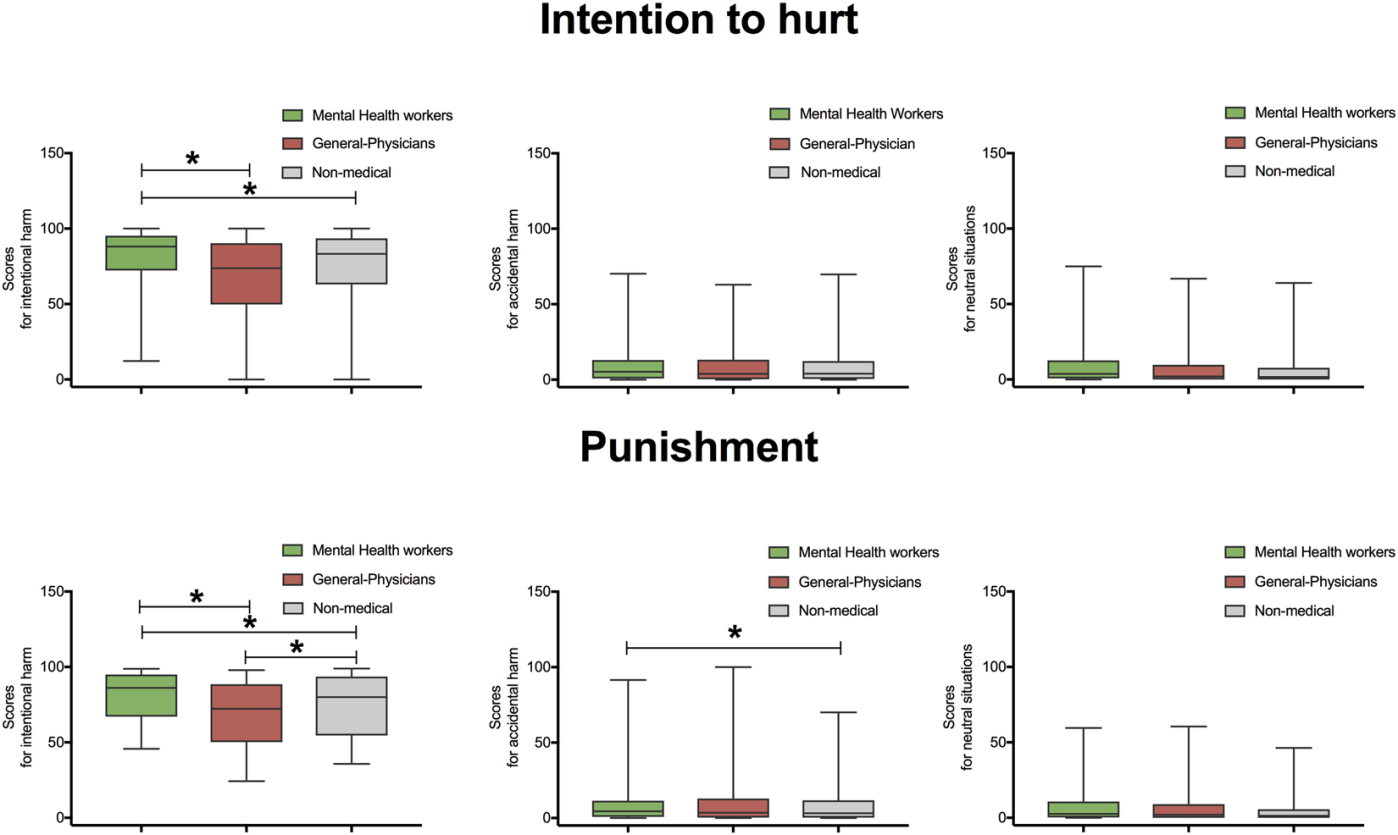
Ratings in intention to hurt and punishment domains in each group. This graph depicts ratings in two empathic measures (Intention to hurt and Punishment) by each condition (intentional and accidental harm and neutral situations) by each group. Stars indicate significant differences (*p* < 0.01).

Regarding intention to hurt, we found a significant interaction between group and condition (*F* (4, 2216) = 7.43, *p* < 0.01, η2 = 0.07). A post-hoc analysis (Tukey HSD, *MS* = 108.76, *df* = 2216) showed that MHWs provided higher ratings for intentional harm than general-physicians (*p* < 0.01) and non-medical workers (*p* < 0.01). No differences were observed between the latter two groups (*p* = 0.31) (see Fig. 2). No other differences were observed in scores for accidental harm and neutral situations between groups (all *p* > 0.2).

For the intention to hurt measure, a triple interaction was observed between condition (intentional harm, accidental harm and neutral situations, group (MHWs, general-physicians and non-medical workers), and years of experience (*F* (2, 2216) = 2.46, *p* = 0.01, η2 = 0.06). A post-hoc analysis (Tukey HSD, *MS* = 108.76, *df* = 2216) showed that in the MHWs, workers with more than 10 years of experience provided lower intention to hurt ratings for intentional harm than workers with less than 10 years of experience (*p* < 0.01). No other contrasts yielded significant effects (no differences were observed neither for general-physicians or non-medical workers (all *p*s > 0.2). Additionally, for this measure a triple interaction emerged between condition, group, and workplace type (*F* (2, 2216) = 3.16, *p* < 0.05, η2 = 0.06). Post-hoc analyses (Tukey HSD, *MS* = 108.76, *df* = 2216) showed that MHWs working in ambulatory environments had higher intention to hurt scores than those working at hospitals (all *p*s < 0.01). Post hoc-analyses did not reveal other significant effects (no differences were observed in general-physicians or in non-medical workers (all *p*s > 0.3).

Finally, regarding the punishment measure, we observed an interaction between group and condition (*F* (4, 2216) = 5.23, *p* < 0.01, η2 = 0.06). A post-hoc analysis (Tukey HSD, *MS* = 108.76, *df* = 2216) showed that MHWs provided higher ratings for intentional harm than general-physicians (*p* < 0.001). In addition, non-medical workers provided higher scores than general-physicians (*p* < 0.01) (see Fig. 2). Moreover, post-hoc analyses revealed that MHWs provided higher punishment scores for accidental harm than non-medical workers (*p* < 0.05).

In addition, for the punishment measure, we observed a triple interaction between condition (intentional harm, accidental harm and neutral situations), group (MHWs, general-physicians, non-medical workers), and years of experience (*F* (2, 2216) = 6.32, *p* = 0.01, η2 = 0.07). A post-hoc analysis (Tukey HSD, *MS* = 108.76, *df* = 2216) showed that MHWs with more than 10 years of experience provided lower punishment ratings than workers with less of 10 years of experience (*p* < 0.01). No other analyses yielded significant effects (no differences were observed in general-physicians or in non-medical workers (all *p*s > 0.42). No interactions were found when workplace type was analyzed.

In sum, we observed higher scores for intentional harm in MHWs than in both other groups. This was the case in various measures, including empathic concern, discomfort, intention to hurt, and punishment. Also, more experienced MHWs presented lower scores in empathy than less-experienced ones. Finally, those working in ambulatory context presented higher empathy scores in discomfort and intention to hurt than those working at hospitals.

### Group differences in empathy measures using IRI scale in a subsample of subjects

In a separate small-scale experiment, we have assessed another group of empathy domains with the interpersonal reactivity index (IRI). To this end, we tested 30 mental health workers, 42 general-physicians, and 28 non-medical participants. We calculated MANOVAs using the empathy domains of IRI as dependent variables (fantasy, EC, PD, and perspective-taking) and group as between factor (MHW, general-physicians and non-medical professionals). Results revealed between-group differences in empathy domains tracked with IRI (Wilk’s Lambda = 0.842) (*F* (8, 150) = 2.52, p < 0.05, η2 = 0.08). This result allowed us to perform additional ANOVAs over each dependent variable of the IRI. A one-way ANOVA revealed that MHWs exhibited higher EC scores than general-physicians and non-medical workers (*F* (2, 77) = 4.07, p < 0.05, η2 = 0.12). The analyses also showed higher perspective-taking scores in MHWs than in other groups (*F* (2, 77) = 2.07, p < 0.05, η2 = 0.06). Analyses over PD (*F* (2, 77) = 1.25, p = 0.27) and fantasy (*F* (2, 77) = 0.58, p = 0.71) scores did not reveal significant differences between groups.

### Moral judgment among groups

#### Impersonal dilemma

**Figure 3:**
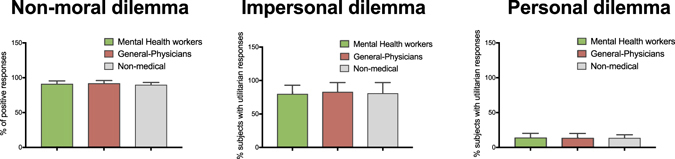
Responses to moral dilemmas in each group. This graph shows percentages of positive responses for the non-moral dilemma, utilitarian and no-utilitarian responses in impersonal and personal dilemmas according to group.

Most participants (929 = 81.9%) delivered a utilitarian response (i.e., yes, flip the switch), and 106 (18.01%) delivered a non-utilitarian response. No significant differences were found between groups (X^2^ (2) = 1.72, *p* = 0.19, Cramer’s V = 0.02) (see Fig. 3).

#### Personal dilemma

A small proportion of participants (158, 13.9%) delivered a utilitarian response (i.e., yes, push the man). Most of them (877, 86.01%) delivered a non-utilitarian response (i.e., no, don’t push the man). No significant differences were found between groups (X^2^ (2) = 1.34, *p* = 0.51, Cramer’s V = 0.02) (see Fig. 3).

#### Non-moral dilemma

A total of 1015 (91.3%) participants provided a positive response to the non-moral dilemma. No significant differences were found between groups (X^2^ (2) = 2.7, *p* = 0.24, Cramer’s V = 0.03) (see Fig. 3).

### The relationship between moral judgment and empathy domains in each group

We analyzed the extent to which moral judgments determine empathy ratings in each group. First, we studied the relationship between empathy and moral judgment in MHWs. To this end, we implemented regression models using each significant empathic measure as a dependent variable, and responses to impersonal and personal dilemmas as independent variables. Models over purpose comprehension for intentional and accidental harm did not reveal significant effects. A multiple regression model using empathic concern ratings for intentional harm (*F* (1, 372) = 3.99, *p* < 0.01, R^2^ = 0.01) showed that responses to the personal dilemma (beta = 1.33, *p* < 0.01, η2 = 0.07) directly explained variance of empathic concern ratings. A model over empathic concern for accidental harm did not yield significant differences.

A new model (*F* (1, 372) = 4.21, *p* < 0.01, R^2^ = 0.02) revealed that responses to the impersonal dilemma (beta = 1.37, *p* < 0.01, η2 = 0.07) directly explained discomfort ratings for intentional harm. Models over discomfort ratings for accidental harm did not reveal significant differences. As regards intention to hurt, a model for intentional harm (*F* (1, 372) = 5.29, *p* < 0.01, R^2^ = 0.02) showed that responses to personal dilemma (beta = 1.42, *p* < 0.01, η2 = 0.08) directly explained intention to hurt ratings. Subjects who provided utilitarian responses to personal dilemma had lower scores in intention to hurt ratings than those with deontological responses.

Regression models for intention to hurt for accidental harm did not reach significant values. Models for punishment ratings for intentional and accidental harm did not reveal significant effects. In sum, results from models revealed an inverse relationship between responses to empathy measures including empathic concern, discomfort, intention to hurt, and utilitarian responses to moral dilemmas.

The relationship between moral judgments and empathy was also analyzed in both control groups (general-physicians and non-medical). Results in general-physicians revealed an inverse relationship between responses to empathic concern and utilitarian responses to impersonal moral dilemmas. Results in non-medical workers did not reach significant differences (for a further description see Supplementary Information section 3.1).

### Factors determining empathy ratings in MHWs

Different regression models were run using each significant empathic measure as a dependent variable and demographic variables (age, gender, years of experience, and workplace type) as independent variables. A first regression model over purpose comprehension ratings (*F* (4, 372) = 0.46, *p* = 0.64, R^2^ = 0.001) yielded no significant effects. The same was true of models analyzing purpose comprehension ratings for accidental harm and neutral situations.

**Figure 4:**
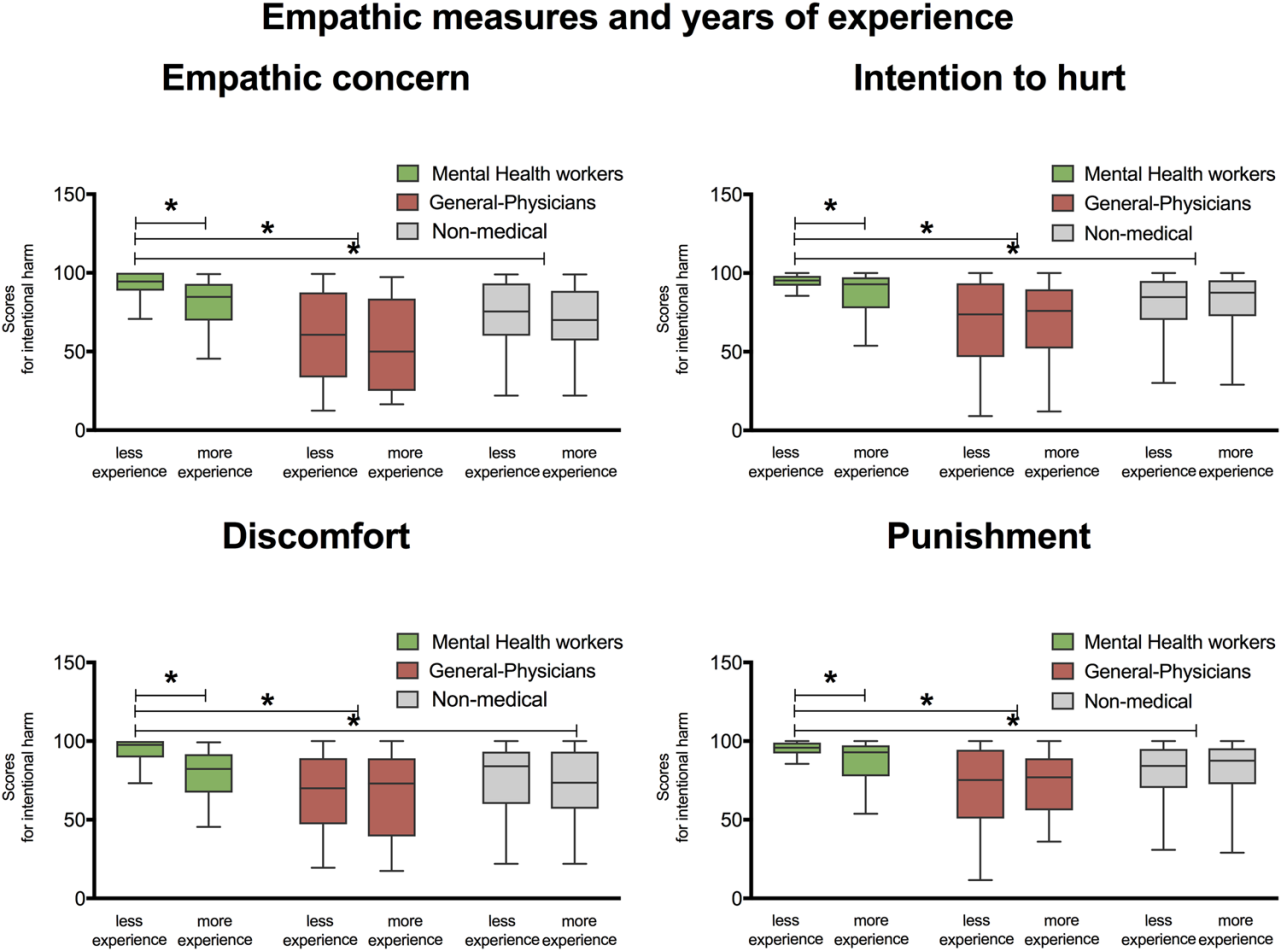
Relationship between empathic domains and years of experience. This graph shows the interaction between empathy measures and years of experience (i.e., less experience: (less than 10 years); high experience: (more than 10 years). Left part of graph shows differences between groups in affective empathy measures: Empathic Concern and Discomfort. The right of the graph shows group differences in moral-cognitive empathy measures: Intention to hurt and Punishment. Stars indicate significant differences at *p* < 0.01.

**Figure 5:**
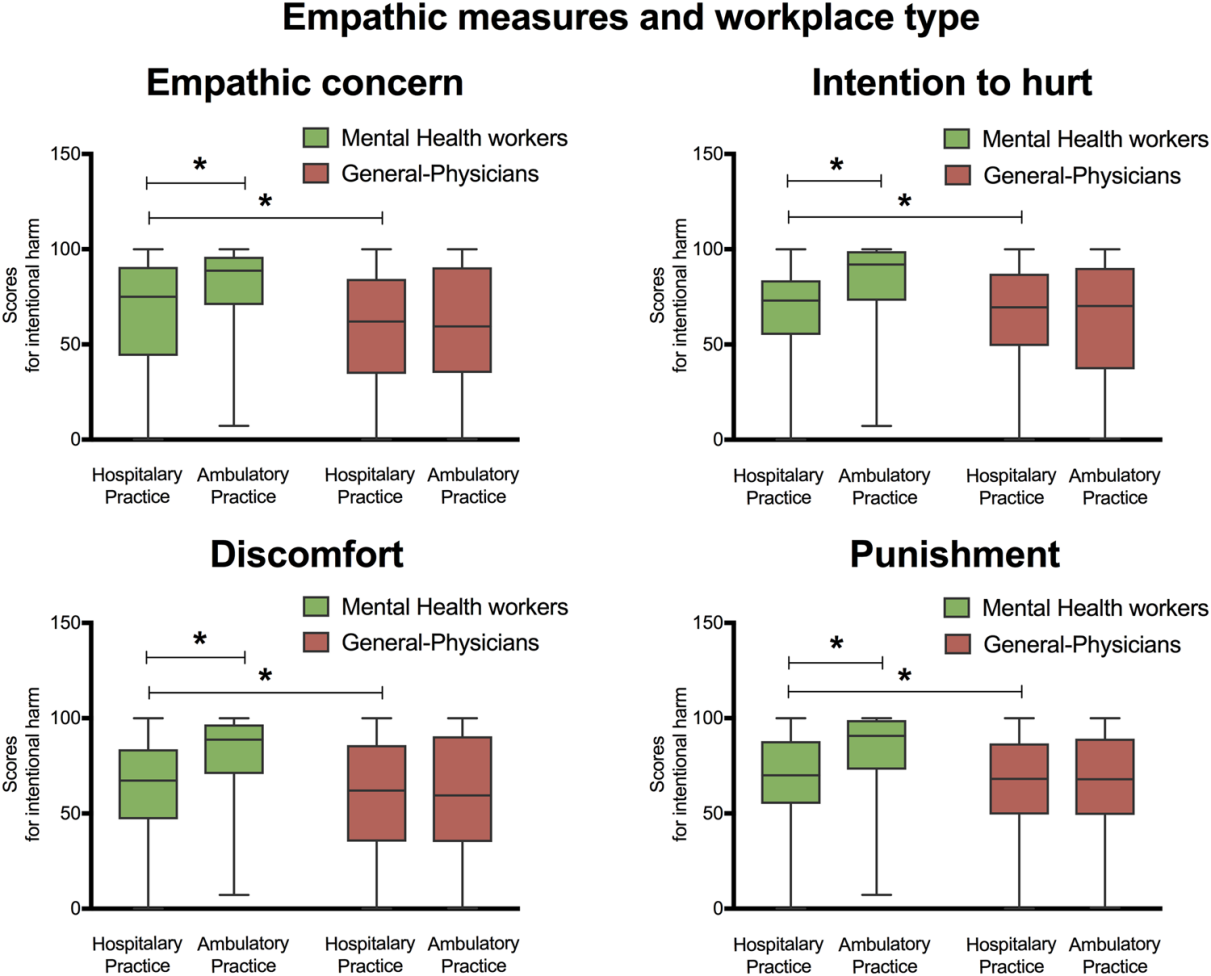
Relationship between empathic domains and workplace type. Graph shows the interaction between empathy measures and workplace type (i.e., hospital practice and ambulatory practice). Left part of graph shows differences between groups in affective empathy measures: Empathic Concern and Discomfort. The right of the graph shows group differences in moral-cognitive empathy measures: Intention to hurt and Punishment. Stars indicate significant differences at *p* < 0.01.

A multiple regression model (*F* (4, 372) = 4.22, *p* < 0.01, R^2^ = 0.08) showed that years of experience (beta = −0.27, *p* < 0.01, η2 = 0.09) and workplace type (beta = −0.17, *p* < 0.05, η2 = 0.06) were associated with empathic concern ratings for intentional harm. Analyses revealed that those subjects with more years of experience (>10 years) had lower empathic concern scores than those with less experience (<10 years). In addition, subjects working at hospitals had lower empathic concern scores than those working in ambulatory settings. No significant differences were revealed by models of empathic concern ratings for accidental harm or for neutral situations (see Figs 4 and 5).

A model over discomfort ratings for intentional harm (*F* (4, 372) = 4.89, *p* < 0.001, R^2^ = 0.09) showed that such a variable was explained by years of experience (beta = −0.22, *p* < 0.001, η2 = 0.07) and workplace type (beta = −0.19, *p* < 0.05, η2 = 0.07). Subjects with more years of experience and working at hospitals had lower discomfort scores. Regression models over discomfort ratings for accidental harm and for neutral situations did not reach significant values (see Figs 4 and 5).

As regards intention to hurt ratings, a multiple regression model (*F* (4, 372) = 5.04, *p* < 0.001, R^2^ = 0.09) showed that years of experience (beta = −0.23, *p* < 0.001, η^2^ = 0.07) and workplace type (beta = −0.21, *p* < 0.01, η^2^ = 0.07) were associated with intention to hurt ratings for intentional harm. An additional model over accidental harm (*F* (4, 372) = 2.38, *p* < 0.05, R^2^ = 0.04) revealed that intention to hurt ratings were explained by workplace type (beta = −0.13, *p* < 0.05, η^2^ = 0.04). Subjects with more years of experience and working at hospitals had lower intention to hurt scores. Models over neutral situations revealed no significant effects (see Figs 4 and 5).

A model over punishment ratings (*F* (4, 372) = 4.58, *p* < 0.001, R^2^ = 0.1) revealed that factors like gender (beta = 0.11, *p* < 0.02, η^2^ = 0.04), years of experience (beta = −0.27, *p* < 0.001, η^2^ = 0.08), and workplace type (beta = −0.17, *p* < 0.01, η^2^ = 0.06) were associated with punishment ratings for intentional harm (see Figs 4 and 5). As in previous measures, the more experienced subjects working at hospitals provided the lowest empathy scores. An additional model over punishment (*F* (4, 372) = 3.18, *p* < 0.05, R^2^ = 0.02) revealed that workplace type (beta = −0.12, *p* < 0.05, η^2^ = 0.04) was associated with ratings for accidental harm. A model for neutral situations did not reveal significant differences. VIF analyses revealed no evidence of multicollinearity between years of experience and age in multiple regression models (VIF values below 1.12).

In sum, for MHWs, years of experience and workplace type were the most crucial factors associated with the ratings for many empathic measures for intentional harm (see Figs 4 and 5).

Results in general-physicians revealed that only years of experience was associated to ratings of empathic concern for intentional harm. In addition, results in the non-medical workers did not reach significant values (for a further description see Supplementary Information section 3.2) (see Figs 4 and 5).

## Discussion

In this population-based study, we examined the empathic profile of MHWs and its relationship with potential modulating factors^19–21^. Relative to general-physicians and non-medical workers, MHWs exhibited higher scores for intentional harm in empathic concern, discomfort, intention to hurt, and punishment measures (see Figs 1 and 2). They were also characterized by higher empathic concern and discomfort scores for accidental harm.

The higher empathy ratings reported by MHWs highlight the distinctive role of empathic skills in their daily practice^1, 26^. Since early stages of training, psychologists and psychiatrists are encouraged to work on their empathic abilities to enhance rapport and improve success in clinical practice^3, 34, 35, 62^. In this sense, the differences observed relative to general-physicians suggest that the fine-tuned empathic profile of MHWs is not merely a reflection of a general effect of clinical experience.

Three major explanations might be listed for these effects. First, empathy scores in MHWs could be a consequence of the differential emphasis placed on particular skills during their training, such as patient comprehension, rapport, and communicative skills. Although empathy skills are developed across medical specialties, students in the mental health field are often more sensitized to the critical role of those communicative and supportive skills for daily practice^29, 63^. Our results are compatible with this possibility, as general-physicians assigned lower scores than MHWs in most empathy measures for intentional and accidental harm. Second, greater empathy in this group could be a response to their constant dealings with human suffering^26, 64^. In the case of MHWs, empathy may be modulated by keeping in touch with others’ pain. This interpretation aligns with evidence that perspective taking and emotional reactivity are modulated by others’ emotional experience^1, 6, 65^. Third, it is also conceivable that people with higher empathic skills are more prone to following a medical career as psychiatrists or psychologists. Moreover, it is plausible that the high empathy skills help MHW in keeping their interest in the profession and achieve longer-running careers. Nevertheless, offering definite accounts of this phenomenon transcends the aims of this study.

Here we observed a positive relationship between EC and discomfort. Some studies show dissociable effects between EC and personal distress (PD)^66–68^, an empathic domain potentially related to discomfort. Along these lines, the degree of EC depends on controlling the degree of pain’ perception of others, i.e., EC depends on how PD is regulated^69, 70^. The inhibition of bottom-up processing of the perception of pain in others can be associated to a major EC in some populations.

Despite of these evidences, we consider that the positive relationship between EC and discomfort in our study is explained by two main reasons. First, our results are in line with studies indicating that PD is a type of vicarious distress, which lead to more prosocial choices and it is necessary to generate intention to help others^5, 67, 71–73^. Evidence of such vicarious distress (PD) is often taken as an indication that subjects are experiencing EC^14, 74–76^. In addition, a positive association between PD and EC is also supported with evidences of neural activations in a reliable ‘empathy for pain’ network, which includes brain areas involved in processing physical pain^14, 74^. Such results indicate an intimate association between self-pain and pain in others^14, 74–76^.

Second, although PD and discomfort are considered self-oriented constructs each of them is measured through different tasks eliciting different emotional-empathic responses. PD has been usually measured with the Interpersonal Reactivity Index (IRI)^57^. PD refers to the disposition to being overwhelmed by intense negative feelings when facing emergency situations (e.g., a natural disaster). This domain has been usually tracked through the Interpersonal Reactivity Index (IRI), which taps self-perception of feelings of anxiety and unease in a group of tense interpersonal settings. By contrast, discomfort refers to the degree of inconformity that subjects feel when facing images of others in pain^77^. In our study discomfort was assessed through the EPT^60, 77^, which explicitly taps the degree of *discomfort* triggered by a painful situation rather than exploring feelings of anxiety or unease facing a situation^60, 77^. The positive relationship we found between EC and discomfort in MHWs replicates previous results^18, 60, 77^. In these studies, both EC and discomfort are considered as affective empathy measures and usually have been reported as enhanced in intentional harms.

Moreover, in an additional small experiment we explored other empathy domains using the IRI scale. Those results revealed higher EC and perspective-taking skills in MHWs relative to general-physicians and non-medical workers. In addition, those analyses did not reveal significant differences in PD scores between groups. Importantly, those results support the explanation that discomfort and PD are different empathic tendencies. Furthermore, it suggests that empathy abilities in MHWs depend on sharing affective experiences, which includes sharing concern and inconformity in presence of other’s suffering. However, empathic skills in MHWs seem to be less related to the degree of anxiety and personal stress that they feel when others face painful situations. These patterns of results should be corroborated in future studies that combine different approaches to assess EC, PD, and discomfort in large-sample populations and in groups where empathy is crucial for daily working.

Crucially, IRI results also showed higher perspective-taking scores in MHWs compared to control groups. Perspective-taking requires a temporary interruption of one’s own point-of-view in an attempt to view a situation as someone else might^4, 78, 79^. Our results suggest that MHWs were more prone to adopting alternative viewpoints, which is understandable given that MHWs need to hone such skills to improve diagnostic and treatment outcomes^26, 80^. These results align with previous studies in social workers showing better treatment results in individuals more capable to assume the needs of others^81^. Higher perspective-taking scores for MHWs may be related with the increased scores observed for this group in intention to hurt and punishment measures of the EPT, which are considered cognitive components of empathy^18, 82^. Together, results from EPT analyses and the IRI scales suggest that MHWs have enhanced affective and cognitive empathy skills.

Previous studies exploring empathy in other medical groups (acupuncturist and internal medicine physicians) have reported a dissociable pattern between EC and PD, suggesting that they might down-regulate their pain response by dampening negative arousal in response to the pain of others and thus freeing up cognitive resources necessary to assist others^69, 70^. This pattern is expected in such groups, because their profession requires inhibiting bottom-up processing of others’ pain to perform well in scenarios marked by physical pain. In contrast, MHWs in our study showed increased EC and discomfort for intentional harms. These results could be explained because empathy is continuously at stake for in MHWs^19, 21, 64^. In this field, empathy is critical during training, in daily clinical practice^34, 35, 63^, and in psychotherapeutic interventions^3^. Thus, while acupuncturists and internal medicine workers can be expected to evidence reductions of anxiety and discomfort for others’ physical pain as an adaptive or coping mechanism, MHWs are likely to develop enhanced experiences of EC and discomfort. Future studies should explore the relationship between EC, PD, EC and discomfort in different medical populations exposed to physical and psychological suffering in others.

Multiple regression analyses revealed that in MHWs and general-physicians empathy dimensions are explained by judgments to moral dilemmas. In particular, responses to the personal dilemma were associated with empathic concern, intention to hurt, and punishment for intentional harm in MHWs. Furthermore, in general-physicians, the responses to the impersonal dilemma were associated with empathic concern. Our results indicate that empathy dimensions are shaped by moral behavior in line with previous studies e.g. refs ^37–39, 83^. The existence of an inverse relationship between affective empathy dimension such as empathic concern and moral judgment has been already reported in medical workers^84, 85^. Our results extend the previous evidences by showing that moral judgment is curved by affective empathy measures including empathic concern and discomfort and by cognitive measures such as intention to hurt and punishment. The interplay between empathy skills and moral judgment seems to be crucial in supporting usual clinical practices in MHWs, including counseling and psychotherapy^19, 52^. Explorations of this assumption transcend our aims and should be further explored in future research.

In addition, we observed that scores in empathy domains were also modulated by years of experience in MHWs. In particular, subjects with more than 10 years of experience showed a reduction of empathy scores for discomfort, intention to hurt, and punishment measures for intentional harm. Although empathic concern scores for intentional harm were also modulated by years of experience in general-physicians, this factor seems to be main determinant for mental health empathy as it was a crucial factor in explaining empathy variance in different empathy domains. Previous studies have reported two types of relationship between empathy and years of experience. While a sort of studies reported that empathic sensitivity decreases in the last stages of medical training^19, 43^, other studies indicated that experienced doctors recover their empathic behavior, an effect explained as a consequence of reduction of personal distress^44, 45^. Our results agree with previous studies that showed an inverse relationship between experience and clinical empathy^13, 43^. Empathy scores in experienced MHWs seem to discard that higher empathy skills are a direct consequence of clinical experience. Conceivably, reductions in empathy scores in MHWs may be a consequence of a progressive desensitization to psychological and mental suffering as they are continuously facing painful situations (see Fig. 4).

Results also revealed that workplace type is a critical in modulating empathy in MHWs. In particular, we observed higher scores of affective empathy measures (i.e., empathic concern and discomfort) in MHWs working at ambulatory environments. This may reflect the comparatively lower quality of care in hospital contexts (see refs ^16, 42, 86^). Indeed, inpatient scenarios are highly stressful, as there, time and privacy for interaction with patients is limited and medical workers usually lack an appropriate environment to favor intimate communication^87^. By contrast, high empathy scores in MHW at ambulatory environments may be due to in these context professionals would have more time to devote attention to their patients^24, 42^ (see Fig. 5).

Environmental conditions seem to play a particularly distinctive role in MHWs, as this factor did not affect empathy ratings in general-physicians. Empathy with psychological suffering could be favored by particular contextual conditions such as intimate, close and warm environments and time to dedicate to others, which are more probably found in ambulatory environments^36, 42^. Changes in affective empathy due to workplace type could suggest that emotional sharing depends on formal circumstances that foster practitioner-patient rapport and closer communication.

The dissimilarities in affective empathic skills in MHWs according to workplace type could have an alternative explanation. It is possible that MHWs working at inpatient environments are more exposed to painful situations than those working at ambulatory settings. Considering this aspect, our results might suggest that MHWs have empathic attitudes that generalize to painful situations irrespective of whether these occur in medical or non-medical contexts. This explanation aligns with evidence from other populations indicating that specific social cognition tendencies, including moral cognition and empathy, go beyond the particular cognitive domain in which they are rooted and generalize to other particular cognitive and situational settings^88, 89^.

Moreover, empathic profile was unaffected by age or gender in MHWs. Notably, the same was true in general-physicians, but not in non-medical workers. As regards age, our results align with previous studies reporting changes in empathy skills mediated by age^6^. Moreover, our results showed that gender modulated empathy scores only in the non-medical group. These results support previous studies^19, 90, 91^ that reported no gender differences when empathy is assessed via experimental approaches, as opposed to self-report measures. The stronger evidence regarding gender differences in empathy has been reported in studies measuring empathy by means of self-report questionnaires e.g., refs ^91–95^. However, such differences are absent when empathy is assessed with experimental tasks^91^ or physiological measures^53, 96^. The fact of age and gender factors counted only for the non-medical group suggests that empathy in MHWs and general-physicians is more mediated by experiential factors such as years of experience and workplace type. Furthermore, our results showed that only in medical groups empathy seems to be more modulated by judgments about the correctness of social actions and moral dispositions.

Our results have important implications to understand how empathy skills are modulated in workers for whom this domain is continuously at stake. Crucially, our results suggest that empathy in MHWs is dynamically sensitive to external influences rather than a static and predetermined social-cognitive skill. The more empathic MHWs seem to be women with less than 10 years of experience working at ambulatory settings and with a more deontological moral profile. Awareness of this pattern could help administrators of medical services to facilitate external conditions that might improve empathy skills in their MHWs. In addition, this knowledge could help to develop educational and assistance plans to those MHWs that have external features associated to reductions of empathy, avoiding negative consequences in patient care.

Second, our results suggest that empathy may be a modifiable cognitive domain even in subjects that use it in their daily working life. Previous initiatives have started to develop plans to improve empathy skills, including affective sharing and perspective taking abilities^88^. Empathy training can impact on social decision-making processes and prosocial behavior^88^, processes which are usually modulated by emotional empathy and perspective-taking skills^97^. Considering empathy as a modifiable factor opens the door to design interventions that may impact on patient care. Future studies should explore which cognitive processes and social factors could favor empathy modifications. In addition, new studies are needed to explore the extent to which changes in empathy skills can foster caring behavior in clinical practice, and whether those changes favor prosocial behavior in other contexts.

Our results seem to deal with a well-documented problem in current science, i.e., the low rates of replicability across studies^98^. The usage of larger samples has been advised for increasing the reproducibility and the precision of estimated effects in psychological studies^98^. Along these lines, our large-scale population-based study gives solidity and allows more robustness the results. Larger samples, as the sample analyzed in this study, increase the statistical power of the estimates concerning social cognitive processes including empathy.

Despite its contributions, our study has some limitations. First, as noted earlier, our work does not determine whether higher empathy skills lead to choosing mental health as a career path or whether they are developed in mental health settings as a consequence of training and sensitization to others’ pain. This consideration should be studied in future research. Second, future population-based studies including larger sets of moral dilemmas, more ecological moral scenarios, self-reported measures, and neurophysiological measures should be explored and related to empathy dimensions in MHWs, to establish the meaningfulness of these findings. Future studies with representative samples from all over the world are needed to determine the generalizability of the present results.

Karl Jaspers introduced empathy as a tool for psychopathological assessment more than a century ago. Jaspers heralded the discovery of empathy as one of most important advances in modern social sciences^33^. This population-based study showed high responses in all empathy domains in MHWs. Furthermore, the empathic profile of these subjects seems sensitive to moral judgment, years of experience, and workplace type. Together, our results indicate that empathy is flexible and modifiable by external factors. Future research should be conducted to elucidate the interaction between empathy and moral decision making in other professions in which good practice hinges on empathy, such as law or social assistance.

## Electronic supplementary material

Supplementary Information
