# Supplementary material for: Empathy for others’ suffering and its mediators in mental health professionals
Source: Sci Rep. 2017 Jul 25;7:6391. doi: 10.1038/s41598-017-06775-y (PMC5527046; doi:10.1038/s41598-017-06775-y)
Supplement: Supplementary file 1 — Supplementary Information [file 41598_2017_6775_MOESM1_ESM.pdf]

Supplementary Information

Empathy for others' suffering and its mediators in mental health professionals

Hernando Santamaría-García[1,2,3,4,5], Sandra Baez[3,4,5,6], Adolfo M. García[4,5,8], Daniel Flichtentrei[7], María Prats[7], Ricardo Mastandueno[7], Mariano Sigman[9], Diana Matallana[10], Marcelo Cetkovich[4,5], Agustín Ibáñez[4, 5,11,12,13*]

1. Supplementary Methods

1.1 Empathy for pain task

We used a modified version of a previously reported EPT [1-6], which evaluates various dimensions of empathy in scenarios featuring intentional and accidental harm. The EPT employed here comprises 11 animated scenarios (4 intentional, 4 accidental, 3 neutral) involving two individuals. Each scenario consists of 3 digital color pictures presented in a sequential manner to simulate motion. Durations of the first, second, and third pictures in each animation were 500, 200, and 1,000 ms, respectively. Three types of situations were depicted: (1) intentional harm, in which one person deliberately inflicts pain on another (e.g., one person purposely steps on someone else's toe); (2) accidental harm, where one person accidentally inflicts pain on another (e.g., one person knocks another individual unintentionally by moving his chair and not realizing that he/she was behind); and (3) control or neutral situations involving no harm (e.g., one hands a flower to another).

Participants were blind to facial emotional reactions as protagonists' faces on pictures were not visible. However, body expressions and postures provided appropriate information about the victim's emotional reaction and the agent's intention. This task evaluates different empathy domains including: (a) comprehension of the accidental or deliberate nature of the action and the intention of the perpetrator to hurt (cognitive, affective, and

moral aspects of empathy), (b) the empathic concern and the degree of discomfort for the victim (affective aspects of empathy), and (c) the punishment ascribed to the perpetrator (moral and cognitive aspects of empathy) (see [1,3]).

In this abbreviated version, participants were asked to respond five questions for each scenario, i.e., (a) purpose comprehension (was the action done on purpose?), (b) empathic concern (how sad do you feel for the victim?), (c) degree of discomfort (how upset do you feel for what happened in the situation?), (d) intention to harm (how bad was the intention?), and (e) punishment (how much penalty does this action deserve?). The question about purpose was answered selecting “Yes” or “No”. The other questions were responded on a visual analogue scale ranging from 0 to 100 –these numbers were not visible to participants. The meaning of the scale extremes depends on the question. For example, in the question “how sad do you feel for the hurt person?”, one extreme of the bar reads “I feel very sad” and the other extreme reads “I don’t feel sad at all”. We measured accuracy for purpose comprehension questions and ratings for the other questions. Previous behavioral [2,3,5,7] and neuroimaging [4,8] studies using similar versions of this task showed that empathy ratings (i.e., empathic concern, discomfort, intention to hurt, and punishment) are higher for intentional than for accidental harm, and ratings for both conditions are higher than those for neutral situations. EPT paradigms reliably induce empathic responses [8-11], engaging putative neural circuits [12] and triggering automatic sensorimotor resonance between self and others [13-15]. Importantly, relative to self-report instruments, these tasks can induce more automatic responses [14,15] –indeed, self-report measures assess the individuals’ beliefs about their experience of empathy and can be biased by social norms and expectations [1,16,17].

1.2 **Moral judgment**

Participants were also presented with two moral dilemmas [18,19], namely, an impersonal one (the standard trolley dilemma) and a personal one (the footbridge dilemma). In the standard trolley dilemma (impersonal), participants had to decide whether they would press a switch to redirect a trolley onto a man to finally save five other individuals. Such a choice is considered a utilitarian response, whereas a refrain from pressing the switch is deemed non-utilitarian. In footbridge dilemma (personal), participants also had the chance to save five people, but this time they were asked whether they would push a man off a bridge in order to stop a trolley from hitting them further down the tracks. Accepting to push the man constitutes a utilitarian response, whereas avoid to do so is considered as a non-utilitarian decision. The impersonal dilemma featured a less emotionally salient harm (e.g., flipping a switch to redirect a trolley onto a man), whereas the personal dilemma featured a more emotionally salient harm (e.g., pushing a man off a bridge) [20,21].

Although both moral dilemmas are equivalent, the impersonal dilemma does not require consideration of an emotion-evoking personal violation to reach a utilitarian outcome [20,21]. Thus, the vast majority of individuals select the utilitarian option for the first case and the non-utilitarian option for the second one [22-24]. Regarding the non-moral dilemma, it is expected that more than 80% of participants provide a positive response [23]. We also included one non-moral dilemma for comparison purposes. In this dilemma participants were asked to choose whether to travel by bus or train given certain time constraints.

2. **Data analysis**

Demographic data were compared among groups with ANOVA tests, except for categorical variables, which were analyzed through X^2 tests. As in previous studies [1-5], the ratings for purpose comprehension, empathic concern, discomfort, intention to hurt and punishment were independently analyzed through a factorial ANOVA, with condition (intentional, accidental, neutral) as a within-subject factor and group (MHWs, general-physicians, non-medical professionals) as a between-subject factor. To explore the extent to which demographic factors modulate responses in empathic measures in each group, we also introduced gender, age, years of experience, and workplace type as covariates. When a significant interaction between group and condition was found, we examined between-group differences in ratings using the Tukey's HSD post-hoc test. Differences among conditions (intentional harm, accidental harm, and neutral situations) for each rating (purpose comprehension, empathic concern, discomfort, intention to hurt, punishment) were examined with Tukey's HSD post-hoc tests. The statistical significance level was set at $p < .05$. Moral judgments were analyzed through Kruskal Wallis test comparisons among groups, and Cramer's V test was used for post-hoc analyses. Between-group differences in responses to the moral dilemmas and the non-moral dilemma were explored using X^2 tests. The statistical significance level was set at $p < .05$.

In addition, we conducted multiple regression analyses to explore whether gender, age, years of experience, workplace type, and responses to moral judgments explained performance on the EPT in each profession. For these analyses, we considered as dependent variables all measures yielding group differences across conditions (intentional, accidental, neutral). We estimated different models in which the empathy measures for intentional and for accidental harm were separately considered as dependent variables. Thus, we ran independent models for purpose comprehension, empathic concern,

discomfort, intention to hurt, and punishment. Different factors including gender, age, workplace type, years of experience and responses to personal and impersonal moral dilemmas were introduced as predictors in all regression models.

Moral judgments, the workplace type and the years of experience were introduced as dummy variables in the regression models. Thus, the moral judgment in the personal and impersonal dilemmas was coded with the number 0 when participant assigned utilitarian responses. Thus, positive values of beta were interpreted as a positive relationship between deontological judgments in moral dilemmas and the empathic measures. For the workplace type, the variable was coded with number 0 when participants have worked mainly in an inpatient environment. Positive values of beta were interpreted as major empathic scores in ambulatory environments. Finally, for the years of experience, the variable was coded with 0 when participant reported less than 10 years of experience. Positive values of beta were interpreted as major empathic scores in subjects with less than 10 years of experience.

3. Supplementary Results

3.1 The relationship between moral judgment and empathy domains in each group

The relationship between moral judgments and empathy was also analyzed in the general-physicians group and in non-medical professionals. In regard to results in general-physicians different regression models were run using each significant empathic measure as a dependent variable and responses to impersonal and personal dilemmas as independent variables. First, models over purpose comprehension for intentional and accidental harm did not reveal significant differences. Models over empathic concern for intentional harm (F (1, 401) = 4.09, p < .01, R^2 = 0.01) revealed that responses to the impersonal dilemma (beta = 1.74, p < .01) were directly related to empathic concern ratings. Models over discomfort, intention to hurt, and punishment for intentional and accidental harm did not

reach significant values. In sum, results from models revealed a possitive relationship between responses to empathic concern and deontological responses to impersonal moral dilemmas. Finally, analyses of the relationship between moral judgment and empathy in non-medical group did not reveal significant differences in any empathy domain.

3.2.1 Factors determining empathy ratings in general-physicians

Regression models over purpose comprehension ratings for harm (intentional, accidental) and neutral situations revealed no significant effects. A regression model over empathic concern ratings (F (4, 401) = 2.8, p < .01, R^2 = 0.08) showed that only the years-of-experience factor (beta = -.1.07, p < .02, η^2 = 0.03) was associated with empathic concern ratings for intentional harm. Models over accidental harm and neutral situations did not reveal significant effects. As regards discomfort, intention to hurt, and punishment ratings, neither models for intentional or accidental harm nor for neutral situations reached significant values. Thus, in general-physicians, only years of experience was associated to ratings of empathic concern for intentional harm (see Figure 4)

3.2.2 Determinants of empathy ratings in non-medical professionals

A first regression model over purpose comprehension ratings did not reveal significant effects. No significant effects were observed for models analyzing purpose comprehension ratings for accidental harm and neutral situations. A regression model over empathic concern (F (4, 372) = 3.04, p < .001, R^2 = 0.09) showed that gender (beta= .11, p < .03, η^2 = 0.04) and age (beta = .13, p < .05, η^2 = 0.09) explained variance of this empathic domain. Models over discomfort, intention to hurt, and punishment for intentional, accidental, and neutral situations did not reach significant values.

References:

1 Baez, S. *et al.* Impairments in negative emotion recognition and empathy for pain in Huntington's disease families. *Neuropsychologia* **68**, 158-167, doi:10.1016/j.neuropsychologia.2015.01.012 (2015).
2 Baez, S. *et al.* Contextual social cognition impairments in schizophrenia and bipolar disorder. *PloS one* **8**, e57664, doi:10.1371/journal.pone.0057664 (2013).
3 Baez, S. *et al.* Primary empathy deficits in frontotemporal dementia. *Frontiers in aging neuroscience* **6**, 262, doi:10.3389/fnagi.2014.00262 (2014).
4 Baez, S. *et al.* Orbitofrontal and limbic signatures of empathic concern and intentional harm in the behavioral variant frontotemporal dementia. *Cortex; a journal devoted to the study of the nervous system and behavior* **75**, 20-32, doi:10.1016/j.cortex.2015.11.007 (2016).
5 Baez, S. *et al.* Integrating intention and context: assessing social cognition in adults with Asperger syndrome. *Frontiers in human neuroscience* **6**, 302, doi:10.3389/fnhum.2012.00302 (2012).
6 Hesse, E. *et al.* Early detection of intentional harm in the human amygdala. *Brain : a journal of neurology* **139**, 54-61, doi:10.1093/brain/awv336 (2016).
7 Gonzalez-Gadea, M. L. *et al.* Emotion recognition and cognitive empathy deficits in adolescent offenders revealed by context-sensitive tasks. *Frontiers in human neuroscience* **8**, 850, doi:10.3389/fnhum.2014.00850 (2014).
8 Decety, J., Michalska, K. J. & Kinzler, K. D. The contribution of emotion and cognition to moral sensitivity: a neurodevelopmental study. *Cerebral cortex* **22**, 209-220, doi:10.1093/cercor/bhr111 (2012).
9 Decety, J. & Cacioppo, S. The speed of morality: a high-density electrical neuroimaging study. *Journal of neurophysiology* **108**, 3068-3072, doi:10.1152/jn.00473.2012 (2012).
10 Treadway, M. T. *et al.* Corticolimbic gating of emotion-driven punishment. *Nature neuroscience* **17**, 1270-1275, doi:10.1038/nn.3781 (2014).
11 Escobar, M. J. *et al.* Brain signatures of moral sensitivity in adolescents with early social deprivation. *Scientific reports* **4**, 5354, doi:10.1038/srep05354 (2014).
12 Akitsuki, Y. & Decety, J. Social context and perceived agency affects empathy for pain: an event-related fMRI investigation. *NeuroImage* **47**, 722-734, doi:10.1016/j.neuroimage.2009.04.091 (2009).
13 Jackson, P. L., Meltzoff, A. N. & Decety, J. How do we perceive the pain of others? A window into the neural processes involved in empathy. *NeuroImage* **24**, 771-779, doi:10.1016/j.neuroimage.2004.09.006 (2005).
14 Jackson, P. L., Rainville, P. & Decety, J. To what extent do we share the pain of others? Insight from the neural bases of pain empathy. *Pain* **125**, 5-9, doi:10.1016/j.pain.2006.09.013 (2006).
15 Singer, T. *et al.* Empathy for pain involves the affective but not sensory components of pain. *Science (New York, N.Y.)* **303**, 1157-1162, doi:10.1126/science.1093535 (2004).
16 Decety, J. The neuroevolution of empathy. *Annals of the New York Academy of Sciences* **1231**, 35-45, doi:10.1111/j.1749-6632.2011.06027.x (2011).
17 Eisenberg, N. & Lennon, R. Sex differences in empathy and related capacities. *Psychological bulletin* **94**, 100 (1983).
18 Thomson, J. The Trolley Problem. *The Yale Law Journal* **94**, 1395-1415 (1985).
19 Foot, P. The problem of abortion and the doctrine of double effect. *Oxford Review* **5**, 5-15 (1967).

20 Greene, J. D., Nystrom, L. E., Engell, A. D., Darley, J. M. & Cohen, J. D. The neural bases of cognitive conflict and control in moral judgment. *Neuron* **44**, 389-400, doi:10.1016/j.neuron.2004.09.027 (2004).

21 Greene, J. D., Sommerville, R. B., Nystrom, L. E., Darley, J. M. & Cohen, J. D. An fMRI investigation of emotional engagement in moral judgment. *Science* **293**, 2105-2108, doi:10.1126/science.1062872 (2001).

22 Ciaramelli, E., Muccioli, M., Ladavas, E. & di Pellegrino, G. Selective deficit in personal moral judgment following damage to ventromedial prefrontal cortex. *Social cognitive and affective neuroscience* **2**, 84-92, doi:10.1093/scan/nsm001 (2007).

23 Geipel, J., Hadjichristidis, C. & Surian, L. The Foreign Language Effect on Moral Judgment: The Role of Emotions and Norms. *PloS one* **10**, e0131529, doi:10.1371/journal.pone.0131529 (2015).

24 Valdesolo, P. & DeSteno, D. Manipulations of emotional context shape moral judgment. *Psychological science* **17**, 476-477, doi:10.1111/j.1467-9280.2006.01731.x (2006).
